# Honours Conferred on Medical Men

**Published:** 1847-04

**Authors:** 


*Honours Conferred on Medical Men.*—The Queen of Spain has created Professor Orfila a Knight of the order of Charles III. Her

Majesty has also made him a dignitary of Spain by a special ordinance. These honours have been conferred on the eminent French Professor in consequence of his having completely remodelled the medical institutions of Madrid. In this reform, Orfila appears to have accomplished a miracle; for he has actually succeeded in satisfying *all* branches of the profession! When we have another medical reform bill under discsusion, it would be desirable to secure his services for Great Britain.

Don Pedro Castello, chief physician of Isabella II. has also been made a Knight of the Order of Charles III. and has had conferred on him the title of *Marquis of Health* (*de la Salud.*) As our French contemporary remarks, this must be a great recommendation to patients, but it is only reasonable to expect that the Esculapian marquis himself will henceforth be free from all attacks of sickness! The creation of this extraordinary title is in accordance with a strange practice prevalent in Spain, of fixing upon some quality or virtue in raising to the peerage an individual who may have no territorial possessions. Hence, among non-professionals, we find Dukes of *Fidelity and Victory;* and for the first time, we believe, among professional men, we have the creation of a Sanitary Marquisate!

Don Pedro Maria Rubio, another eminent Spanish physician, has been created a Knight of the Royal American Order of Isabella the Catholic.—*Ibid.*

